# Systemic and intratumoral balances between monocytes/macrophages and lymphocytes predict prognosis in hepatocellular carcinoma patients after surgery

**DOI:** 10.18632/oncotarget.9049

**Published:** 2016-04-27

**Authors:** Rui Liao, Ning Jiang, Zhuo-Wei Tang, De-Wei Li, Ping Huang, Shi-Qiao Luo, Jian-Ping Gong, Cheng-You Du

**Affiliations:** ^1^ Department of Hepatobiliary Surgery, the First Affiliated Hospital of Chongqing Medical University, Chongqing 400016, China; ^2^ Department of General Surgery, Mianyang Central Hospital, Mianyang 621000, China; ^3^ Chongqing Key Laboratory of Hepatobiliary Surgery, The Second Affiliated Hospital of Chongqing Medical University, Chongqing 400010, China; ^4^ Department of Hepatobiliary Surgery, the Second Affiliated Hospital of Chongqing Medical University, Chongqing 400010, China

**Keywords:** hepatocellular carcinoma, neutrophil, monocyte, macrophage, lymphocyte

## Abstract

The peripheral neutrophil-monocyte/lymphocyte ratio (NMLR) and intratumoral CD16/CD8 ratio (iMLR) may have prognostic value in hepatocellular carcinoma (HCC) patients after curative resection. In this study, the circulating NMLR was examined 387 HCC patients who underwent curative resection between 2006 and 2009. Intratumoral levels of CD4, CD8, CD16 and CD68 and the CD16/CD8 ratio were determined immunohistologically. The prognostic values of clinicopathological parameters, including NMLR and iMLR, were evaluated. NMLR was predictive of overall survival (OS) and recurrence-free survival (RFS) when patients in the training cohort (*n* = 256) were separated into high (> 1.2) and low (≤ 1.2) NMLR subgroups. NMLR was also an independent predictor of low alpha-fetoprotein (AFP) expression and early recurrence. High NMLR was associated with increases in clinicopathological variables, including alanine aminotransferase (ALT), tumor number, tumor size and BCLC stage. In addition, iMLR strongly predicted risk of recurrence and patient survival, and was positively correlated with NMLR. These findings were confirmed in an independent validation patient cohort (*n* = 131). Peripheral NMLR and iMLR may thus be useful prognostic markers, and anti-inflammatory treatment may be beneficial in HCC patients after curative hepatectomy.

## INTRODUCTION

Hepatocellular carcinoma (HCC) is a malignant tumor with high incidence worldwide [1]. Currently, although multiple options exist for HCC patients, surgery is still the mainstay of treatment. However, the recurrence rate is approximately 50%–75% within 5 years after resection [2]. Thus, it is necessary to identify patients with a high risk of recurrence for increased monitoring and to make appropriate treatment-related decisions.

The causes of the high recurrence rate in HCC are complex and multifactorial. Two of the most significant factors affecting recurrence are the tumor-promoting effects of chronic inflammation and the malignant biological behaviors of cancer cells [3, 4]. Most HCC patients have a history of chronic liver disease, mainly induced by hepatitis B or C viral infection. In these patients, the accumulation of inflammatory cells likely contributes to the malignant potential of cancer cells and HCC formation [5]. In turn, some hepatic inflammatory/immune cells, such as tumor-associated macrophages and cancer-associated fibroblasts, are activated by tumor cells, further enhancing tumor phenotypes, proliferation, angiogenesis, and invasion [6–8].

Recently, clinical and experimental evidence has demonstrated that both the systemic and focal inflammatory responses in an inflamed liver might promote the formation of tumors and consequently influence the prognosis of HCC patients [3, 9, 10]. In HCC microenvironments, tumor-related leukocytes, especially activated monocytes, can trigger and polarize T-cell responses and promote inflammation-induced tumor development [11]. Moreover, circulating monocytes have the ability to mobilize and migrate to liver tissues in response to inflammation or tumor environmental signals, where they can then further differentiate into tissue macrophages and dendritic cells [12, 13]. Accumulating evidence shows that crosstalk between monocytes/macrophages and other inflammatory/immune cells (e.g. hepatic stellate cells and lymphocytes) can promote tumorigenesis and angiogenesis via inflammatory signatures [11, 14]. Lymphocytes also play a crucial role in HCC progression through immunoselection in an immunosuppressive network, which dictates immune responses to tumors [15]. Based on these factors, some inflammatory/immune cell counts, such as monocyte counts [16], lymphocyte counts [17], and ratios, such as neutrophil-lymphocyte ratio (NLR) [18], were reported to predict recurrence and survival in HCC. We also recently identified preoperative NLR as a simple prognostic marker for patients with single-nodule small HCC after curative resection [19]. Lymphocyte-monocyte ratio (MLR) has also been used to predict prognosis in various cancers [20–23]. Here, we developed an integrated indicator derived from peripheral neutrophil, monocyte, and lymphocyte (neutrophil and monocyte to lymphocyte ratio, NMLR) levels to predict the outcomes of HCC after curative resection.

In this study, we investigated the association between systemic inflammation and focal infiltration of inflammatory cells (including intratumoral CD4^+^, CD8^+^, CD16^+^, and CD68^+^ cells, and CD16/CD8 ratio) and the recurrence of HCC and clinical outcomes. Our data suggested that circulating NMLR and intratumoral CD16/CD8 ratio were useful biomarkers for HCC prognosis and may provide a better understanding of the impact of inflammation on tumors.

## RESULTS

### Baseline characteristics

The baseline characteristics of patients are described in Table 1. In the training cohort, the median follow-up time was 44 months (range: 1.5–84 months). The 1-, 3-, and 5-year OS rates were 85.7%, 61.3% and 43.2%, and RFS rates were 80.6%, 49.2% and 37.8%, respectively. The median age of the 214 male and 42 female patients was 53 years.

**Table 1:** Characteristics of patients in the training and validation cohorts

Characteristics		Training Cohort (*n* = 256)	Validation Cohort (*n* = 131)	*P*
Age (year)	≤ 50 > 50	107 (41.8%) 149 (58.2%)	62 (47.3%) 69 (52.7%)	0.330
Gender	Female Male	42 (16.4%) 214 (83.6%)	18 (13.7%) 113 (86.3%)	0.554
ALT (U/L)	≤ 40 > 40	136 (53.1%) 120 (46.9%)	69 (52.7%) 62 (47.3%)	1.000
Liver cirrhosis	Yes No	223 (87.1%) 33 (12.9%)	123 (93.9%) 8 (6.1%)	0.054
HBsAg	Positive Negative	208 (81.2%) 48 (18.8%)	116 (88.5%) 15 (11.5%)	0.080
AFP (ng/ml)	≤ 20 > 20	102 (39.8%) 154 (60.2%)	46 (35.1%) 85 (64.9%)	0.379
Platelet count (109/L)	≤ 100 > 100	56 (21.9%) 200 (78.1%)	40 (30.5%) 91 (69.5%)	0.081
Tumor number	Single Multiple	222 (86.7%) 34 (13.3%)	118 (90.1%) 13 (9.9%)	0.412
Vascular invasion	Yes No	81 (31.6%) 175 (68.4%)	18 (13.7%) 113 (86.3%)	**< 0.001**
Tumor differentiation	I–II III–IV	192 (75.0%) 64 (25.0%)	95 (72.5%) 36 (27.5%)	0.624
Tumor encapsulation	Yes No	141 (55.1%) 115 (44.9%)	65 (49.6%) 66 (50.4%)	0.333
Tumor size (cm)	≤ 5.0 > 5.0	172 (67.2%) 84 (32.8%)	88 (67.2%) 43 (32.8%)	1.000
TNM stage	I I-II	177 (69.1%) 79 (30.9%)	87 (66.4%) 44 (33.6%)	0.645
BCLC stage	0–A B–C	119 (46.5%) 137 (53.5%)	70 (53.4%) 61 (46.6%)	0.199

Abbreviations: ALT: alanine aminotransferase; HBsAg: hepatitis B surface antigen; AFP: alpha fetoprotein; TNM: tumor node metastasis; BCLC: Barcelona clinic liver cancer.

In the validation cohort, the median follow-up period was 36.9 months (range: 4–60). The cumulative survival and recurrence (in brackets) rates at 1-, 3-, and 5-years were 84.3% (78.4%), 62.1% (50.2%), and 44.3% (38.6%), respectively. The clinicopathological characteristics of the validation cohort were similar to the training cohort, with the exception of vascular invasion (Table 1).

### Correlation of NMLR with prognosis in the training cohort

**Figure 1:**
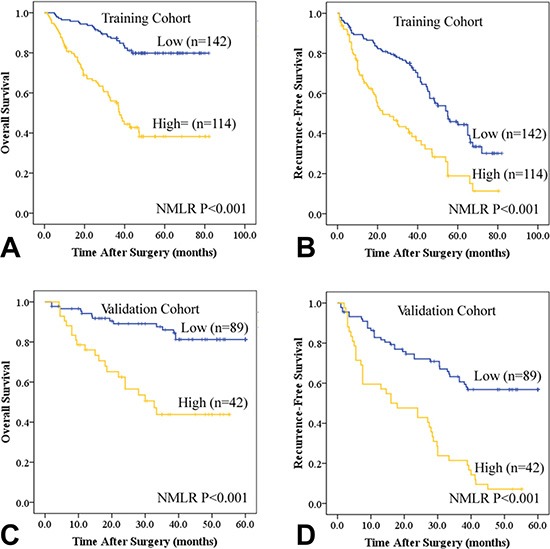
Kaplan-Meier estimates of recurrence-free survival (RFS) and overall survival (OS) based on peripheral neutrophil and monocyte to lymphocyte ratio (NMLR) in HCC patients after curative resection in the training (A and B) and validation cohorts (C and D).

In univariate analyses of our data, AFP, HBsAg, platelet counts, tumor multiplicity, tumor differentiation, tumor size, vascular invasion, TNM stage, and BCLC stage were prognostic indicators of OS and/or RFS (Table 2). Kaplan-Meier analysis indicated that patients with high NMLR scores had shorter OS (median, 23 months) and RFS (median, 18 months) than those with low NMLR scores (median, 41 and 30 months, respectively) (Figure 1). Both high NLR and MLR scores were associated with poor OS (median, 27 and 24 months, respectively) and RFS (median, 21 and 19 months, respectively) (Figure S1). Significant clinical factors were then used for further multivariate analyses. MLR and NMLR showed higher predictive values for both OS (*P* < 0.001, HR = 0.128 and *P* < 0.001, HR = 19.307, respectively) and RFS (*P* = 0.006, HR = 0.389 and *P* < 0.001, HR = 4.457), respectively. Platelet counts, tumor number, tumor size, vascular invasion, and TNM stage were independent predictors for OS only (*P* < 0.001, HR = 0.357; *P* = 0.001, HR = 2.600; *P* < 0.001, HR = 2.996; *P* = 0.025, HR = 1.695; and *P* = 0.002, HR = 2.107, respectively). Additionally, HBsAg, AFP, and BCLC stage were associated with RFS alone (*P* = 0.006, HR = 1.916; *P* = 0.012, HR = 1.575; and *P* = 0.001, HR = 1.837, respectively). Based on RFS, recurrence was divided into early recurrence (≤ 24 months, *n* = 81) and late recurrence (> 24 months, *n* = 61). In univariate analyses, patients with high NMLR scores were more likely to suffer from early tumor recurrences (*P* = 0.012 for univariate analyses and *P* = 0.019 for multivariate analyses) (Table S1). In addition, the prognostic value of NMLR also applied to patients negative for AFP in stratified analyses (Figure S2).

**Table 2:** Univariate and multivariate analyses of prognostic factors in the training cohort (*n* = 256)

Factors	RFS			OS		
	Univariate	Multivariate		Univariate	Multivariate	
	*P*	HR (95% CI)	*P*	*P*	HR (95% CI)	*P*
Age, year(≤ 50 vs > 50)	0.094		NA	0.371		NA
Gender(Female vs Male)	0.769		NA	0.787		NA
ALT, U/L(≤ 40 vs > 40)	0.042		0.172	0.235		NA
Liver cirrhosis(Yes vs No)	0.404		NA	0.246		NA
HBsAg(Positive vs Negative)	**0.007**	1.916 (1.207–3.042)	**0.006**	**0.043**		0.104
AFP, ng/ml(≤ 20 vs > 20)	**0.005**	1.575 (1.107–2.240)	**0.012**	0.090		NA
Platelet count, 10^9^/L(≤ 100 vs > 100)	0.151		NA	**0.008**	0.357 (0.215–0.592)	**< 0.001**
Tumor encapsulation(Yes vs No)	0.196		NA	0.263		NA
Tumor number(Single vs Multiple)	**0.036**		0.308	**0.011**	2.600 (1.461–4.627)	**0.001**
Vascular invasion(Yes vs No)	**< 0.001**		0.272	**0.001**	1.695 (1.068–2.692)	**0.025**
Tumor differentiation(I–II vs III–IV)	**0.037**		0.205	**0.047**		0.341
Tumor size, cm(≤ 5.0 vs > 5.0)	**< 0.001**		0.163	**< 0.001**	2.996 (1.895–4.737)	**< 0.001**
TNM stage(I vs II–III)	**0.012**		0.065	**< 0.001**	2.107 (1.329–3.342)	**0.002**
BCLC stage(0/A vs B/C)	**< 0.001**	1.837 (1.283–2.632)	**0.001**	**< 0.001**		0.175
NLR(≤ 2.5 vs > 2.5)	**0.004**		0.882	**< 0.001**		0.636
MLR(≤ 0.3 vs > 0.3)	**< 0.001**	0.389 (0.199–0.760)	**0.006**	**< 0.001**	0.128 (0.060–0.270)	**< 0.001**
NMLR(≤ 1.2 vs >1.2)	**< 0.001**	4.457 (2.254–8.812)	**< 0.001**	**< 0.001**	19.307 (8.804–42.341)	**< 0.001**
Intratumoral CD16	**< 0.001**	1.457 (1.008–2.106)	**0.045**	**0.020**		0.418
Intratumoral CD8	**0.001**		0.057	**0.030**		0.506
IntratumoralCD16/CD8(≤ 1.1 vs > 1.1)	**< 0.001**	1.869 (1.285–2.718)	**< 0.001**	**0.023**	1.876 (1.174–2.998)	**0.009**

Univariate analysis: Kaplan-Meier method; multivariate analysis: Cox proportional hazards regression model. Abbreviations: RFS: Recurrence-Free Survival; OS: overall survival; ALT: alanine aminotransferase; HBsAg: hepatitis B surface antigen; AFP: alpha fetoprotein; TNM: tumor-nodes-metastasis; NLR: neutrophil to lymphocyte ratio; MLR: monocyte to lymphocyte ratio; NMLR: neutrophil and monocyte to lymphocyte ratio; NA: not adopted.

### Validating the prognostic value of NMLR

For further validation, we investigated the predictive value of NMLR in an additional set of 131 HCC patients (Table 1 and Table S2). Similar to the training cohort, univariate analysis revealed that increased NMLR score was associated with poor OS (*P* < 0.001) and RFS (*P* < 0.001) (Table S2 and Figure 1). Both NLR and MLR were related to HCC prognosis (Table S2 and Figure S1). Multivariate analyses suggested that NMLR was a powerful prognostic marker for survival (HR = 4.403, 95% CI = 2.104–9.215, *P* < 0.001) and recurrence (HR = 3.044, 95% CI = 1.864–4.972, *P* < 0.001) (Table S2). NMLR also had prognostic value in patients with early recurrence and AFP-negative subgroups (Table S1 and Figure S2).

### Association of NMLR with clinicopathologic features and inflammation-associated parameters

In the training cohort, we found that NMLR scores > 1.2 correlated with elevated ALT (*P* = 0.017), tumor multiplicity (*P* = 0.015), large tumor size (*P* = 0.001), and advanced BCLC stage (*P* < 0.001) (Table S3). NLR scores > 2.5 were associated with higher platelet counts (*P* = 0.004) (Table S4). MLR was associated with sex (*P* = 0.041), tumor number (*P* = 0.015), tumor size (*P* = 0.002), TNM (*P* = 0.041), and BCLC stage (*P* < 0.001) (Table S4). Associations between these scores and clinicopathologic features in the validation cohort are shown in Tables S3 and S4.

To better understand the clinical meaning of NMLR as a prognostic inflammation index, we compared the relationship between NMLR and some inflammation-associated parameters, including NLR, MLR, C-reactive protein (CRP), Glasgow prognostic score (GPS), prognostic index (PI), and prognostic nutritional index (PNI) [24]. NMLR was positively correlated with NLR (*r* = 0.513 and 0.689, both *P* < 0.001), MLR (*r* = 0.858 and 0.714, both *P* < 0.001), GPS (*r* = 0.129 and 0.168, *P* = 0.039 and 0.042, respectively) and PNI (*r* = 0.180 and 0.221, *P* = 0.004 and 0.011, respectively) in both cohorts (Table S5).

### The prognostic significance of immunostaining parameters

**Figure 2:**
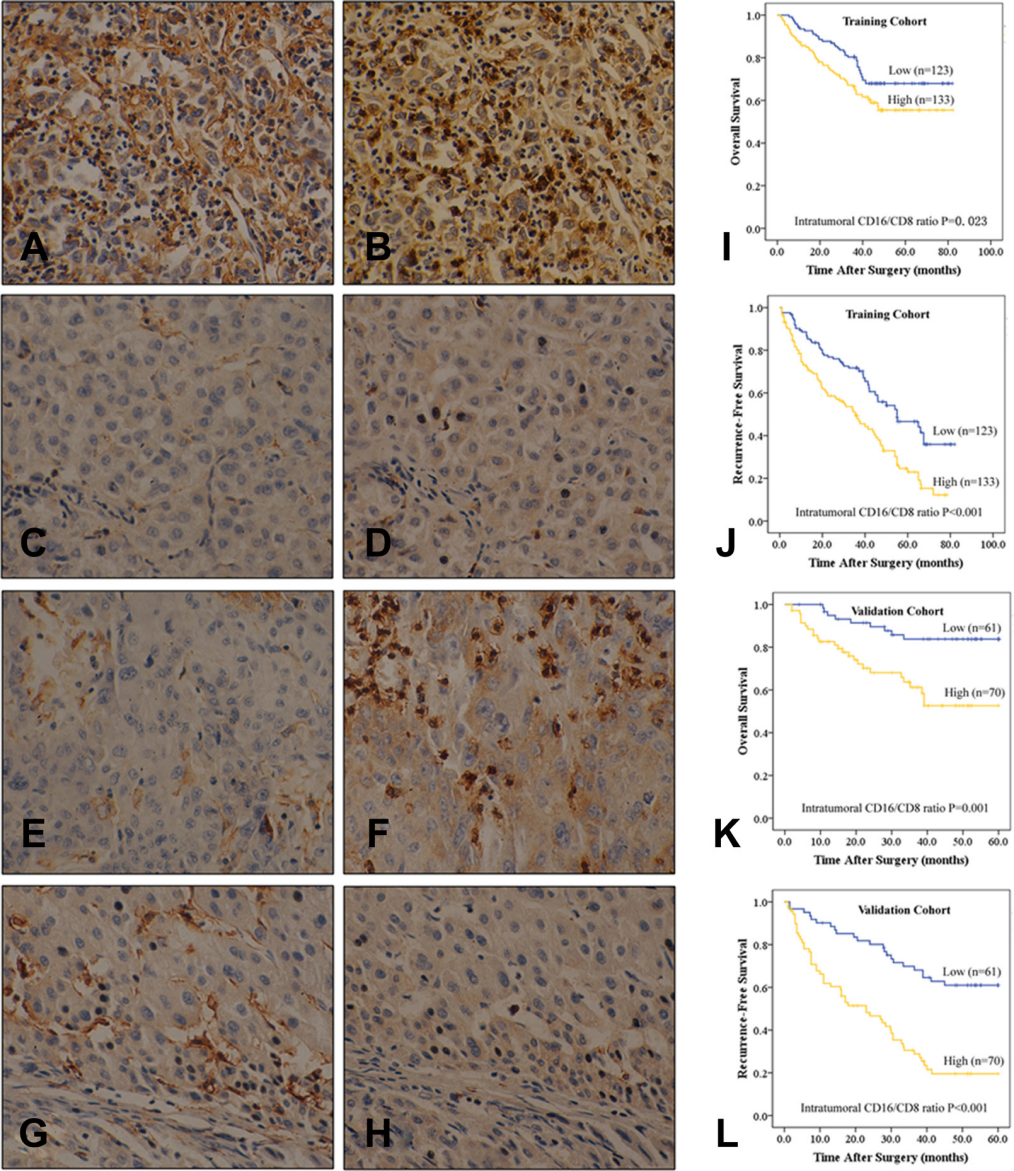
Immunohistochemical and kaplan-meier analyses of intratumoral CD16 and CD8, and the ratio of the two Consecutive sections were used for immunohistochemical staining of intratumoral CD16- (A, C, E, and F) and CD8- (B, D, F, and H) positive cells, which were divided into four subgroups: (**A** and **B**) both high; (**C** and **D**) both low; (**E** and **F**) low CD16 and high CD8 expression; (**G** and **H**) high CD16 and low CD8 expression (400× magnification). (**I**–**L**) Overall survival (OS, I and K) and recurrence-free survival (RFS, J and L) based on intratumoral CD16/CD8 ratio in HCC patients after curative resection in the training (I and J) and validation cohorts (K and L).

We quantified intratumoral CD16^+^ and CD68^+^ cell and lymphocyte (CD4 and CD8) numbers and found that the CD16^+^ cell to CD8^+^ lymphocyte ratio (iMLR) was indicative of immune imbalance in the local microenvironment (Figure 2A–2H and Figure S3). In both the training and validation cohorts, levels of intratumoral CD8 and CD16 cells and iMLR strongly predicted the risk of recurrence and/or patient survival (Table 2, Table S2, Figure 2I–2L and Figure S4). In addition, elevated iMLR correlated with high BCLC stage (*P* = 0.033 and 0.035, respectively) in both cohorts (Table S6). Intratumoral CD4 and CD68 levels were not associated with prognosis in HCC patients.

### The correlation between intratumoral CD16^+^ cell to CD8^+^ lymphocyte ratio and systemic NMLR

**Figure 3:**
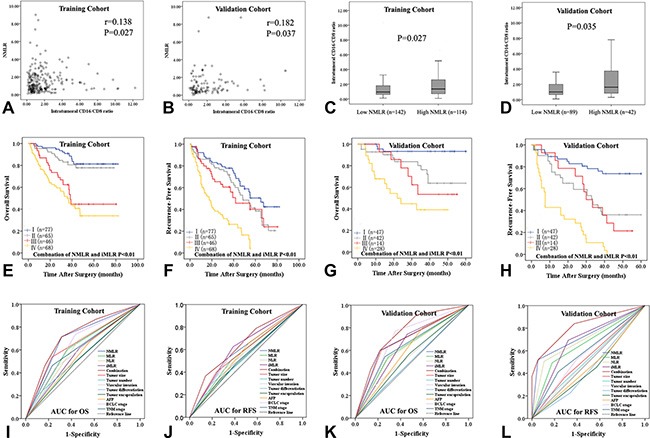
**The correlation between peripheral neutrophil and monocyte to lymphocyte ratio (NMLR) and intratumoral CD16/CD8 ratio (iMLR) in the training (A) and validation cohorts (B).** (**C** and **D**) Intratumoral CD16/CD8 ratio in the low and high NMLR subgroups in both cohorts. (**E**–**H**) Low CD16 with high CD8 was associated with both prolonged survival (E and G) and decreased recurrence (F and H). (**I**–**L**) The predictive ability of combined NMLR and iMLR was compared to other inflammatory/immune cell ratios and other established tumor prognostic variables by receiver operating characteristics (ROC) curves.

In both cohorts, scatter plot analyses showed a positive correlation between iMLR and systemic NMLR (*r* = 0.138, *P* = 0.027 and *r* = 0.182, *P* = 0.037) (Table 3, Figure 3A and 3B). Intratumoral CD16/CD8 ratio was higher in the high NMLR group than in the low NMLR group (1.60 ± 0.49 vs 1.46 ± 0.50, *P* = 0.027; 1.67 ± 0.477 vs 1.47 ± 0.502, *P* = 0.035) (Figure 3C and 3D). The combination of iMLR and NMLR scores (both low vs both high, Figure 3E–3H) predicted OS and RFS (both *P* < 0.001) better than either measure alone (Figure 3E–3H), and better than other established tumor prognostic variables such as tumor size, tumor differentiation, vascular invasion, and BCLC/TNM stage (Figure 3I–3L, Table S7).

**Table 3:** Correlation between intratumoral CD16/CD8 and peripheral parameters (NLR, MLR and NMLR)

Variable	Intratumoral CD16/CD8							
	Training Cohort (*n* = 256)				Validation Cohort (*n* = 131)			
	Mean	SD	*r*	*P*	Mean	SD	*r*	*P*
**NLR**	3.22	3.18	0.036	0.565	2.85	2.72	0.016	0.858
**MLR**	0.39	0.33	0.122	0.051	0.32	0.22	0.124	0.158
**NMLR**	1.45	1.28	0.138	**0.027**	1.16	1.35	0.182	**0.037**
**Intratumoral CD16/CD8**	1.77	1.89	NA	NA	1.93	1.94	NA	NA

Abbreviations: NLR: neutrophil-lymphocyte ratio; MLR: monocyte to lymphocyte ratio; NMLR: neutrophil and monocyte to lymphocyte ratio.

## DISCUSSION

Accumulating evidence suggests that cancer cells can upregulate inflammatory processes that subsequently impact patient survival in various cancers [6, 13]. Meanwhile, pretreatment levels of peripheral neutrophils, lymphocytes, monocytes, and focal infiltrating inflammatory cells are thought to be predictive of HCC prognosis [9, 10, 16–18]. Here, we constructed an integrated prognostic score that combined circulating levels of neutrophils, monocytes, and lymphocytes (NMLR), and found it was an independent predictor of survival in HCC patients after hepatectomy. Compared to NLR, MLR and other tumor characteristics such as BCLC stage, tumor size, and vascular invasion, NMLR predicted outcomes more accurately in HCC patients after surgery. Therefore, this novel integrated prognostic score might be useful for monitoring HCC recurrence according to characteristics of individual tumors.

To the best of our knowledge, this is the first study investigating the predictive value of circulating MLR and NMLR for clinical HCC outcomes. The present study demonstrates that the balance of neutrophils/monocytes and lymphocytes in systemic inflammatory response is related to patient survival. The roles of these cells in tumorigenesis might explain the predictive power of these measures for prognosis. Growing evidence suggests that activated neutrophils exhibit considerable tumor-promoting activities in response to environmental pro-tumor signals and via cellular crosstalk with tumor cells [25, 26]. For example, neutrophils contribute to the initiation of monocyte recruitment by various mechanisms [13]. Moreover, both monocytes/macrophages and neutrophils are involved in the regulation of immune responses in various inflammatory and tumor microenvironments [27]. NMLR might therefore reflect the complex interaction and potential synergistic effects between monocyte/macrophages and neutrophils in tumor microenvironments. Furthermore, monocytes promote tumorigenesis by producing multiple immunosuppressive, tumor-promoting chemokines/cytokines [28]. Lymphocytes also release cytokines and chemokines, such as IL-16, CCL21, and VEGFA, that attract monocytes, dendritic cells (DCs), and endothelial cells to the tumor core and invasive margin [29]. Thus, complex interplay between inflammatory/immune cell populations and resulting synergistic or opposing effects may differentially affect tumor growth. When peripheral lymphocytes dominate, patients may have relatively desirable outcomes; conversely, when circulating neutrophils/monocytes dominate, patients might have worse prognoses.

Peripheral blood monocytes can be subdivided into different subpopulations based on surface CD14 and CD16 expression. Previously, we found that CD14 was almost absent in cancerous HCC tissues [30]. However, the expression and predictive roles of intratumoral CD16 in HCC are unknown. Here, histological examination revealed that high intratumoral CD16 and low intratumoral CD8 expression were associated with poor prognosis in HCC patients. In most tumors, tumor-activated macrophages differentiate from circulating monocytes, and their acquired physiologies and resulting phenotypes contribute to angiogenesis, which promotes tumor growth, invasiveness, and migration [31–34]. Many studies have described the anti-cancer activities of CD8^+^ T cells [35–37]. Furthermore, tumor-infiltrating monocytes/macrophages may induce apoptosis of activated CD8^+^ T cells, thereby suppressing their proliferation and activation within the tumor bed [38–40]. In addition, tumor-induced senescence (TIS)-CD8^+^ T cells suppress lympho-proliferative response, and massive reductions in lymphocyte numbers may result in an insufficient immunological reaction to the tumor. Conversely, TIS-CD8^+^ T cells promote CD16^+^ expression in monocytes/macrophages and the production of pro-inflammatory cytokines (TNF, IL-1β, and IL-6) and angiogenic factors (MMP-9, VEGF-A, and IL-8), which can affect tumor progression [41]. Thus, the intratumoral balance between CD16^+^ cells and CD8^+^ T cells affects inflammatory/immune responses and outcome in HCC patients. Moreover, our immunohistochemical analysis showed that a high intratumoral CD16^+^ cell-to-CD8^+^ T cell ratio was associated with higher circulating NMLR. Because human CD16^+^ cells are precursors of inflammatory tissue macrophages and inflammatory DCs and localize to chronically inflamed and fibrotic liver tissues [42], intratumoral CD16/CD8 ratio may reflect the balance between different inflammatory/immune cell populations involved in tumor development. Additionally, increased production of monocytes/macrophages, DCs, and natural killer (NK) cells (focal inflammation) may mirror increases in circulating monocyte levels (systemic inflammation) and reflect high tumor burdens. Thus, immunotherapies that activate CD8^+^ T cells may be effective adjuvant treatments for HCC. Comprehensive systemic treatments also need to be carefully evaluated even after the tumor is removed.

Here, we found that the density of intratumoral CD16, but not CD68, was related to overall survival and recurrence in HCC. The diversity, plasticity, and polarization of macrophages (M1 and M2 phenotypes) in the tumor microenvironment may contribute to this discrepancy [34]. M1 cells are CD14- and CD86-expressing macrophages involved in active microbial killing. In contrast, M2 cells express CD16 and CD163 and are associated with tissue remodeling and tumor progression. In response to polarization signals, such as IL-4, IL-13, transforming growth factor-β, and matrix metalloproteinase-9, monocytes in the tumor polarize into M2 macrophages [34, 43]. The M2 phenotype seems to dominate in tumor-associated macrophages that act as “protumoral macrophages.” Therefore, macrophages that express CD16 may orchestrate various aspects of tumor progression and accurately predict clinical outcomes in HCC. However, this effect and the underlying mechanisms need to be clarified in future studies.

AFP is the most widely used indicator for HCC diagnosis and treatment, although 30% to 40% of HCC patients have normal AFP levels following surgery [44]. Here, our data indicate that elevated NMLR predicted poor prognosis in patients with normal AFP, and these patients may require more extensive follow-ups after surgery. In clinical practice, it is challenging to predict early recurrence (≤ 24 months), which represents a true metastasis [45]. Encouragingly, NMLR may help predict early recurrence. The relationship between elevated NMLR and poor prognosis also suggests that systemic inflammatory response promotes the dissemination of primary HCC tumor cells. In this regard, anti-inflammatory treatment may be beneficial in the management of HCC.

In conclusion, we have demonstrated that the combination of NMLR and iMLR was predictive of outcome after curative resection in two independent HCC patient cohorts. Our data also suggest that systemic inflammatory response is indicative of concurrent focal inflammation in tumors. The balance between monocytes/macrophages and lymphocytes in the tumor milieu influences the prognosis of HCC after resection. These results show that NMLR and iMLR, which are easy to measure, are effective for monitoring HCC prognosis and may help optimize the selection of anti-inflammatory therapies in clinical practice. However, further studies are necessary to investigate the molecular mechanisms of crosstalk between various inflammatory/immune cells (e.g. monocytes/macrophages, neutrophil, and lymphocytes) in HCC.

## MATERIALS AND METHODS

### Patients and specimens

Between January 2006 and December 2008, a retrospective study was conducted in an independent cohort including a total of 256 consecutive archived patient records. A total of 131 consecutive HCC patients between January and December 2009 were selected as a validation cohort. The inclusion and exclusion criteria were the same as our previous report [30]. All patients received curative resection of HCC at the First Affiliated Hospital of Chongqing Medical University. Archival specimens and blood samples were obtained after informed consent. This study protocol and ethical approval for the use of human subjects were obtained from the Ethics Review Committee of the First Affiliated Hospital of Chongqing Medical University. The baseline clinical characteristics of all patients are described in Table 1.

### Follow-up postoperative treatment

Postoperatively, all patients had follow-ups every 1 to 6 months after operation and were monitored prospectively by serum alpha-fetoprotein (AFP) and abdominal computed tomography (CT) or/and magnetic resonance imaging (MRI) examination. Follow-ups were completed in December 2014. Recurrence-free survival (RFS) was defined as the interval between surgery and the first confirmed recurrence. Overall survival (OS) was the interval between the first operation and death or the last monitoring time point for surviving patients. In one month after surgery, patients with recurrence at risk (e.g. vascular invasion and spreading nodules) were treated by transcatheter arterial chemoembolization with triple chemotherapeutic agents (oxaliplatin, epirubicin, and irinotecan). If recurrence was suspected due to typical imaging appearance and an elevated AFP level, additional detection procedures, such as hepatic angiography, were performed; the treatment modality varied among individuals.

### Neutrophil and monocyte to lymphocyte ratio (NMLR)

Neutrophil and monocyte to lymphocyte ratio (NMLR) was calculated as follows: NMLR = preoperative peripheral neutrophil (N) X monocyte (M) / lymphocyte (L) counts. Here, a “minimum *p* value” approach was used to estimate an optimal cut-off for NMLR values to best separate of patients based on RFS using X-tile software (Yale University, New Haven, CT) as described in our previous report [19]. The optimal cut-off point for NMLR was 1.2. The similarly calculated cut-off values for NLR, MLR, and intratumoral CD16/CD8 were 2.5, 0.3 and 1.1, respectively.

### Tissue microarray and immunohistochemistry

A tissue microarray (TMA) was constructed as described previously [9]. Liver tissue sections were deparaffinized, hydrated, and washed. After antigen retrieval, immunohistochemical staining was performed using a 2-step protocol with the Envision+ system and DAB kit (DAKO). Primary antibodies were anti-human monoclonal antibodies combined with CD68 (1:100; ab955, Abcam), CD4 (1:100; sc-59032, Santa Cruz), CD8 (1:100; sc-1181, Santa Cruz), and CD16 (1:100; sc-20052, Santa Cruz). Under high-power magnification (400X), micrographs of five independent microscopic fields of stained cells were screened and captured using a Leica DMLA light microscope (Leica Microsystems, Wetzlar, Germany). Data are expressed as mean values of the triplicate cores from each patient.

### Statistical analysis

All statistical analyses were performed with SPSS 16.0 (SPSS, Inc., Chicago, IL). Continuous variables were summarized as the mean ± SD and compared by Student's *t*-tests or non-parametric Mann-Whitney U-tests. For the comparison of categorical variables, χ^2^ or Fisher's exact tests were used as appropriate. Correlations between variables were analyzed using Pearson's or Spearman's ρ coefficient tests.

Survival curves were analyzed using the Kaplan-Meier method and compared by the log-rank test. Univariate and multivariate analyses were calculated using the multivariate Cox proportional hazard regression model. The best cut-off values for NLR, MLR, MNLR, and iMLR were determined using X-tile software (Yale University, New Haven, CT). Sensitivity and specificity were defined by applying receiver operating characteristics (ROC) curves. Two-tailed *P*-values < 0.05 were considered statistically significant.

## SUPPLEMENTARY MATERIAL FIGURES AND TABLES


