# Supplementary material for: Systemic and intratumoral balances between monocytes/macrophages and lymphocytes predict prognosis in hepatocellular carcinoma patients after surgery
Source: Oncotarget. 2016 Apr 27;7(21):30951–61. doi: 10.18632/oncotarget.9049 (PMC5058730; doi:10.18632/oncotarget.9049)
Supplement: Supplementary file 1 [file oncotarget-07-30951-s001.pdf]

Systemic and intratumoral balances between monocytes/ macrophages and lymphocytes predict prognosis in hepatocellular carcinoma patients after surgery

Supplementary Materials

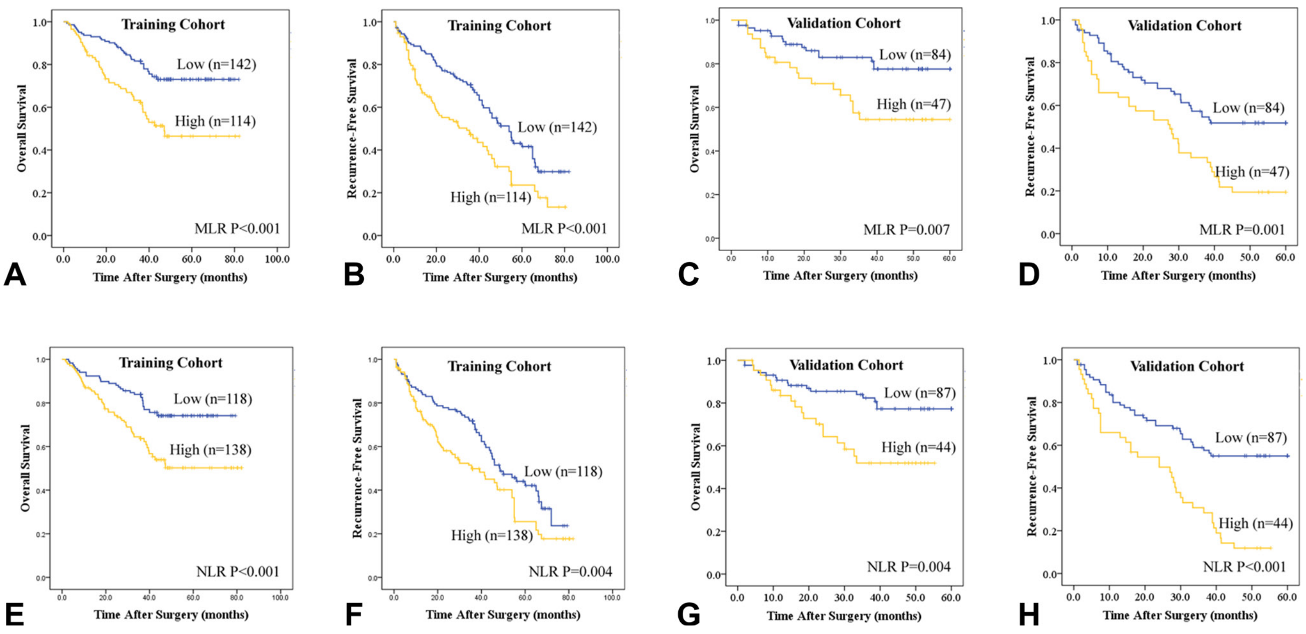

Supplementary Figure S1: Comparison of overall survival (OS, A, C, E, and G) and recurrence-free survival (RFS, B, D, F, and H) based on peripheral monocyte to lymphocyte ratio (MLR, A–D) and neutrophil to lymphocyte ratio (NLR, E–H) in the training (A, B, E, and F) and validation cohorts (C, D, G, and H).

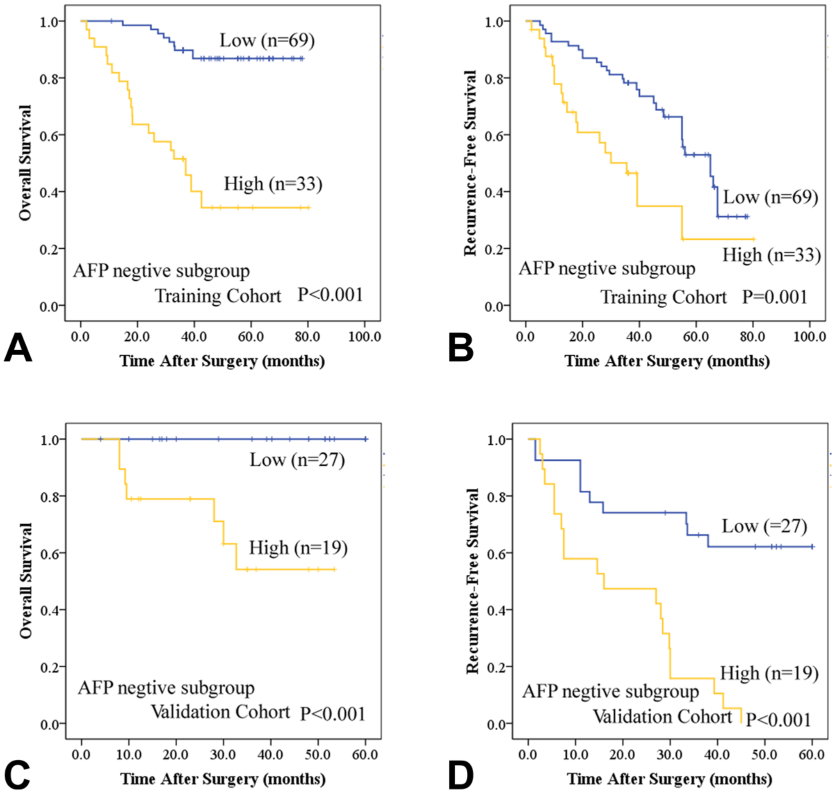

Supplementary Figure S2: Kaplan-Meier analyses of overall survival (OS, A and C) and recurrence-free survival (RFS, B and D) based on peripheral neutrophil and monocyte to lymphocyte ratio (NMLR) in the negative AFP subgroup in the training (A and B) and validation cohorts (C and D).

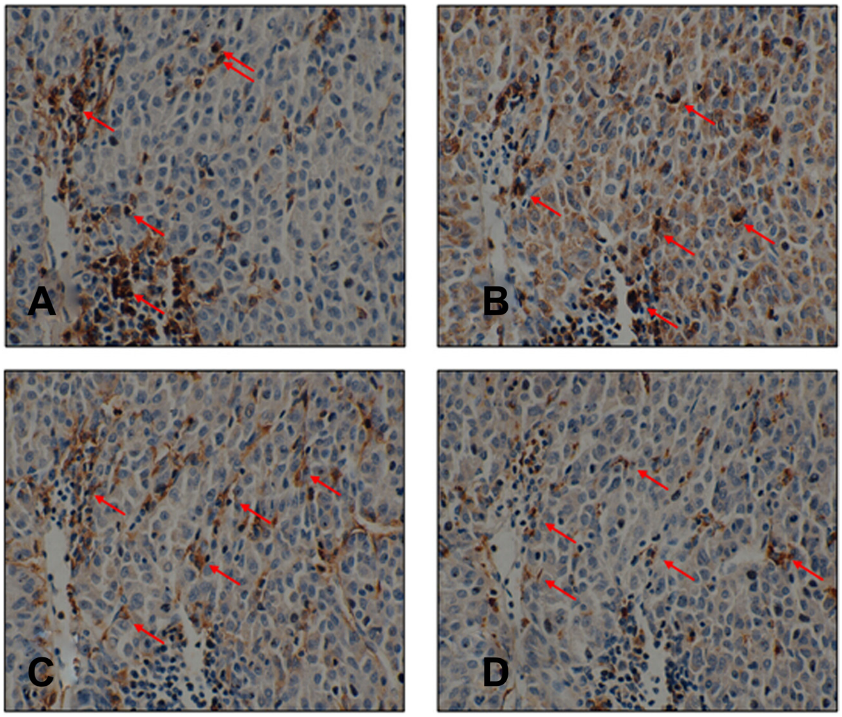

Supplementary Figure S3: Immunohistochemical staining of intratumoral CD4 (A), CD8 (B), CD16 (C), and CD68 (D) in a representative case. Positive cells are stained brown (red arrows, 200×).

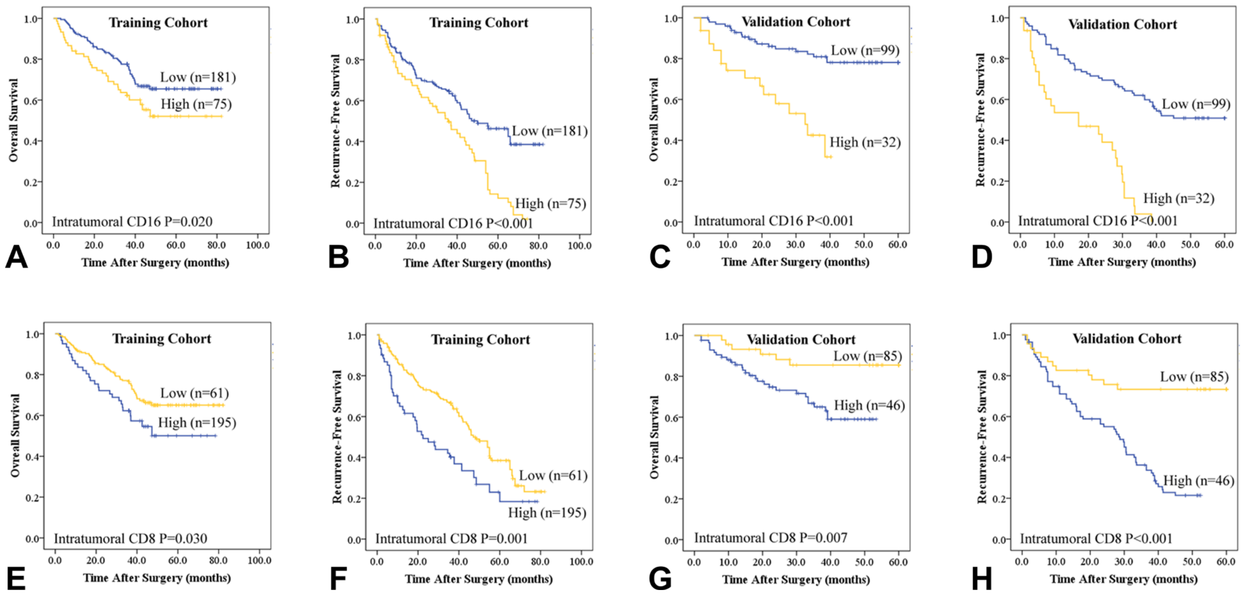

Supplementary Figure S4: Prognostic significance of CD16 and CD8 in HCC patients undergoing resection. Kaplan-Meier analyses showed the overall survival (OS, A, C, E, and G) and recurrence-free survival (RFS, B, D, F, and H) based on CD16 (**A–D**) and CD8 (**E–H**) in the training (A, B, E, and F) and validation cohorts (C, D, G, and H).

Supplementary Table S1: Univariate and multivariate analyses for early recurrence subgroups

Factors	Training Cohort (*n* = 81)			Validation Cohort (*n* = 48)		
	Univariate	Multivariate		Univariate	Multivariate	
	P	**HR (95% CI)**	***P***	***P***	**HR (95% CI)**	***P***
Tumor size, cm (≤ 5.0 vs > 5.0)	**0.05**		0.666	0.135		NA
Tumor number (Single vs Multiple)	0.105		NA	**0.016**		0.131
Tumor differentiation (I–II vs III–IV)	0.144		NA	**< 0.001**	0.017 (0.000–0.843)	**0.041**
BCLC stage (0/A vs B/C)	**0.025**	1.971 (1.039–3.742)	**0.038**	**0.015**		0.052
NLR (≤ 2.5vs > 2.5)	0.684		NA	**0.017**		0.189
MLR (≤ 0.3 vs > 0.3)	0.142		NA	**0.05**		0.183
NMLR (≤ 1.2 vs > 1.2)	**0.012**	2.072 (1.126–3.811)	**0.019**	**< 0.001**	5.478 (1.848–16.238)	**0.010**

Univariate analysis: Kaplan-Meier method; multivariate analysis: Cox proportional hazards regression model. Abbreviations: HR: Hazard Ratio; BCLC: Barcelona clinic liver cancer; NLR: neutrophil to lymphocyte ratio; MLR: monocyte to lymphocyte ratio; NMLR: neutrophil and monocyte to lymphocyte ratio; NA: not adopted.

Supplementary Table S2: Univariate and multivariate analyses of prognostic factors in the validation cohort (*n* = 131)

**Factors**	**RFS**			**OS**		
	**Univariate**	**Multivariate**		**Univariate**	**Multivariate**	
	P	**HR (95% CI)**	***P***	***P***	**HR (95% CI)**	***P***
Age, year (≤ 50 vs > 50)	0.234		NA	0.055		NA
Gender (Female vs Male)	0.645		NA	0.323		NA
ALT, U/L (≤ 40 vs > 40)	0.477		NA	0.340		NA
Liver cirrhosis (Yes vs No)	0.151		NA	0.733		NA
HBsAg (Positive vs Negative)	0.530		NA	0.621		NA
AFP, ng/ml (≤ 20 vs > 20)	0.508		NA	0.055		NA
Platelet count, 109/L (≤ 100 vs > 100)	0.192		NA	0.033		0.161
Tumor encapsulation (Yes vs No)	0.361		NA	0.335		NA
Tumor number (Single vs Multiple)	0.446		NA	0.771		NA
Vascular invasion (Yes vs No)	**0.008**	2.515 (1.257–5.030)	**0.009**	0.001	6.408 (2.526–16.254)	**< 0.001**
Tumor differentiation (I–II vs III–IV)	0.212		NA	0.877		NA
Tumor size, cm (≤ 5.0 vs > 5.0)	**0.012**	2.192 (1.332–3.610)	**0.002**	**< 0.001**	6.154 (2.982–12.699)	**< 0.001**
TNM stage (I vs II–III)	**0.006**		0.951	**0.001**		0.801
BCLC stage (0/A vs B/C)	**0.004**		0.192	**< 0.001**		0.988
NLR (≤ 2.5 vs > 2.5)	**< 0.001**		0.064	**0.004**		0.699
MLR (≤ 0.3 vs > 0.3)	**0.001**		0.916	**0.007**		0.790
NMLR (≤ 1.2 vs > 1.2)	**< 0.001**	3.044 (1.864–4.972)	**< 0.001**	**< 0.001**	4.403 (2.104–9.215)	**< 0.001**
Intratumoral CD16	**< 0.001**	3.422 (2.018–5.803)	**< 0.001**	**< 0.001**	2.407 (1.118–5.179)	**0.025**
Intratumoral CD8	**< 0.001**	0.412 (0.217–0.784)	**0.007**	**0.007**		**0.624**
Intratumoral CD16/CD8 (≤ 1.1 vs > 1.1)	**< 0.001**		0.068	**0.001**	2.337 (1.018–5.364)	**0.045**

Univariate analysis: Kaplan-Meier method; multivariate analysis: Cox proportional hazards regression model. Abbreviations: RFS: Recurrence-Free Survival; OS: overall survival; ALT: alanine aminotransferase; HBsAg: hepatitis B surface antigen; AFP: alpha fetoprotein; TNM: tumor-nodes-metastasis; NLR: neutrophil to lymphocyte ratio; MLR: monocyte to lymphocyte ratio; NMLR: neutrophil and monocyte to lymphocyte ratio; NA: not adopted.

Supplementary Table S3: Correlation between NMLR and clinicopathological characteristics of patients in the training and validation cohorts

Characteristics		NMLR					
		Training Cohort (n = 256)			Validation Cohort (n = 131)		
		**≤ 1.2**	**> 1.2**	***P***	**≤ 1.2**	**> 1.2**	***P***
Age (year)	≤ 50<br>> 50	61<br>81	46<br>68	0.703	42<br>47	20<br>22	1.000
Gender	Female<br>Male	21<br>121	21<br>93	0.498	6<br>83	12<br>30	**0.002**
ALT (U/L)	≤ 40<br>> 40	85<br>57	51<br>63	**0.017**	50<br>39	19<br>23	0.265
Liver cirrhosis	Yes<br>No	123<br>19	100<br>14	0.853	83<br>6	40<br>2	1.000
HBsAg	Positive<br>Negative	110<br>32	98<br>16	0.107	76<br>13	40<br>2	0.142
AFP (ng/ml)	≤ 20<br>> 20	63<br>79	39<br>75	0.123	27<br>62	19<br>23	0.118
Platelet count (109/L)	≤ 100<br>> 100	31<br>111	25<br>89	1.000	28<br>61	13<br>29	1.000
Tumor number	Single<br>Multiple	130<br>12	92<br>22	**0.015**	80<br>9	38<br>4	1.000
Vascular invasion	Yes<br>No	41<br>101	40<br>74	0.344	13<br>76	5<br>37	0.790
Tumor differentiation	I/II<br>I/II	111<br>31	81<br>33	0.195	61<br>28	34<br>8	0.149
Tumor encapsulation	Yes<br>No	83<br>59	58<br>56	0.256	47<br>42	18<br>24	0.350
Tumor size (cm)	≤ 5.0<br>> 5.0	108<br>34	64<br>50	**0.001**	62<br>27	26<br>16	0.427
TNM stage	I<br>II/III	105<br>37	72<br>42	0.077	65<br>24	22<br>20	**0.029**
BCLC stage	0/A<br>B/C	82<br>60	37<br>77	**< 0.001**	50<br>39	20<br>22	0.453

Abbreviations: NMLR: neutrophil and monocyte to lymphocyte ratio; ALT: alanine aminotransferase; HBsAg: hepatitis B surface antigen; AFP: alpha fetoprotein; TNM: tumor node metastasis; BCLC: Barcelona clinic liver cancer.

Supplementary Table S4: Correlation between NLR and MLR and clinicopathological characteristics of patients in the training and validation cohorts

Characteristics		NLR						MLR					
		Training Cohort (*n* = 256)			Validation Cohort (*n* = 131)			Training Cohort (*n* = 256)			Validation Cohort (*n* = 131)		
		**≤ 2.5**	**> 2.5**	***P***	**≤ 2.5**	**> 2.5**	***P***	**≤ 0.3**	**> 0.3**	***P***	**≤ 0.3**	**> 0.3**	***P***
Age (year)	≤ 50<br>> 50	49<br>69	58<br>80	1.000	43<br>44	19<br>25	0.580	44<br>70	63<br>79	0.374	40<br>44	22<br>25	1.000
Gender	Female<br>Male	15<br>103	27<br>111	0.176	79<br>8	34<br>10	0.057	25<br>89	17<br>125	**0.041**	76<br>8	37<br>10	0.070
ALT (U/L)	≤ 40<br>> 40	59<br>59	77<br>61	0.381	46<br>41	23<br>21	1.000	54<br>60	82<br>60	0.103	48<br>36	21<br>26	0.203
Liver cirrhosis	Yes<br>No	102<br>16	121<br>17	0.852	81<br>6	42<br>2	0.717	103<br>11	120<br>22	0.192	77<br>7	46<br>1	0.258
HBsAg	Positive<br>Negative	91<br>27	117<br>21	0.148	75<br>12	41<br>3	0.384	97<br>17	111<br>31	0.198	13<br>71	2<br>45	0.083
AFP (ng/ml)	≤ 20<br>> 20	48<br>70	54<br>84	0.898	27<br>60	19<br>25	0.180	41<br>73	61<br>81	0.304	26<br>58	20<br>27	0.189
Platelet count (109/L)	≤ 100<br>> 100	16<br>102	40<br>98	**0.004**	27<br>60	14<br>30	1.000	23<br>91	33<br>109	0.649	25<br>59	16<br>31	0.695
Tumor number	Single<br>Multiple	104<br>14	118<br>20	0.583	80<br>7	38<br>6	0.359	92<br>22	130<br>12	**0.015**	74<br>10	44<br>3	0.376
Vascular invasion	Yes<br>No	79<br>39	96<br>42	0.687	12<br>75	6<br>38	1.000	42<br>72	39<br>103	0.137	14<br>70	4<br>43	0.290
Tumor differentiation	I-II<br>III-IV	89<br>29	103<br>35	1.000	60<br>27	35<br>9	0.221	82<br>32	110<br>32	0.314	59<br>25	36<br>11	0.541
Tumor encapsulation	Yes<br>No	65<br>53	76<br>62	1.000	43<br>44	22<br>22	1.000	59<br>55	82<br>60	0.377	43<br>41	22<br>25	0.716
Tumor size (cm)	≤ 5.0<br>> 5.0	84<br>34	88<br>50	0.231	59<br>28	29<br>15	0.846	65<br>49	107<br>35	**0.002**	56<br>28	32<br>15	1.000
TNM stage	I<br>II-III	82<br>36	95<br>43	1.000	59<br>28	28<br>16	0.697	71<br>43	106<br>36	**0.041**	62<br>22	25<br>22	**0.021**
BCLC stage	0/A<br>B/C	61<br>57	58<br>80	0.133	48<br>39	22<br>22	0.584	38<br>76	81<br>61	**< 0.001**	43<br>41	27<br>20	0.584

Abbreviations: NLR: neutrophil to lymphocyte ratio; MLR: monocyte to lymphocyte ratio; ALT: alanine aminotransferase; HBsAg: hepatitis B surface antigen; AFP: alpha fetoprotein; TNM: tumor node metastasis; BCLC: Barcelona clinic liver cancer.

Supplementary Table S5: Correlation between NMLR and other inflammation associated parameters

Parameters		NMLR (≤ 1.2 vs > 1.2)					
		Training Cohort (*n* = 256)			Validation Cohort (*n* = 131)		
		n	*r*	*p*	*n*	*r*	*P*
CRP	≤ 10 mg/L<br>> 10 mg/L	232<br>24	0.089	0.154	114<br>17	0.095	0.282
NLR	≤ 2.5<br>> 2.5	118<br>138	0.513	< 0.001	87<br>44	0.689	< 0.001
MLR	≤ 0.3<br>> 0.3	114<br>142	0.858	< 0.001	84<br>47	0.714	< 0.001
GPS	0<br>1/2	231<br>25	0.129	0.039	111<br>20	0.168	0.042
PI	0<br>1/2	232<br>24	0.061	0.329	114<br>17	0.075	0.392
PNI	0<br>1	220<br>36	0.180	0.004	114<br>17	0.221	0.011

Abbreviations: NMLR: neutrophil and monocyte to lymphocyte ratio; CRP: C-reactive protein; NLR: neutrophil to lymphocyte ratio; MLR: monocyte to lymphocyte ratio; GPS: Glasgow prognostic score; PI: prognostic index; PNI: prognostic nutritional index.

Supplementary Table S6: Correlation between iMLR and clinicopathological characteristics of patients in the training and validation cohorts

Characteristics		iMLR					
		Training Cohort (*n* = 256)			Validation Cohort (*n* = 131)		
		≤ 1.1	> 1.1	*P*	≤ 1.1	> 1.1	*P*
Age (year)	≤ 50<br>> 50	52<br>71	55<br>78	0.900	24<br>37	38<br>32	0.114
Gender	Female<br>Male	22<br>101	20<br>113	0.613	52<br>9	61<br>9	0.803
ALT (U/L)	≤ 40<br>> 40	67<br>56	69<br>64	0.708	33<br>28	36<br>34	0.861
Liver cirrhosis	Yes<br>No	112<br>11	111<br>22	0.092	60<br>1	63<br>7	0.067
HBsAg	Positive<br>Negative	103<br>20	105<br>28	0.341	55<br>6	61<br>9	0.784
AFP (ng/ml)	≤ 20<br>> 20	50<br>73	52<br>81	0.898	16<br>45	30<br>40	0.066
Platelet count (109/L)	≤ 100<br>> 100	32<br>91	24<br>109	0.133	22<br>39	19<br>51	0.345
Tumor number	Single<br>Multiple	108<br>15	114<br>19	0.713	56<br>5	62<br>8	0.574
Vascular invasion	Yes<br>No	34<br>89	47<br>86	0.226	7<br>54	11<br>59	0.613
Tumor differentiation	I-II<br>III-IV	91<br>32	101<br>32	0.773	42<br>19	53<br>17	0.435
Tumor encapsulation	Yes<br>No	68<br>55	73<br>60	1.000	29<br>32	36<br>34	0.727

Tumor size (cm)	≤ 5.0<br>> 5.0	85<br>38	87<br>46	0.595	45<br>16	43<br>27	0.142
TNM stage	I<br>II-III	83<br>40	94<br>39	0.591	47<br>14	40<br>30	**0.026**
BCLC stage	0/A<br>B/C	66<br>57	53<br>80	**0.033**	39<br>22	31<br>39	**0.035**

Abbreviations: iMLR: intratumoral CD16/CD8 ratio; ALT: alanine aminotransferase; HBsAg: hepatitis B surface antigen; AFP: alpha fetoprotein; TNM: tumor node metastasis; BCLC: Barcelona clinic liver cancer.

Supplementary Table S7: The area under the receiver operating characteristic curve (AUROC) of patients in the training and validation cohorts

**Characteristics**		**AUROC (95% CI)**			
		**Training Cohort (*n* = 256)**		**Validation Cohort (*n* = 131)**	
		**RFS**	**OS**	**RFS**	**OS**
AFP (ng/ml)	≤ 20<br>> 20	0.568<br>(0.497–0.639)	0.557<br>(0.483–0.632)	0.458<br>(0.359–0.558)	0.603<br>(0.498–0.708)
Tumor number	Single<br>Multiple	0.533<br>(0.462-0.603)	0.552<br>(0.475–0.629)	0.524<br>(0.425–0.624)	0.510<br>(0.397–0.623)
Vascular invasion	Yes<br>No	0.564<br>(0.493–0.634)	0.592<br>(0.517–0.668)	0.511<br>(0.411–0.611)	0.562<br>(0.447–0.678)
Tumor differentiation	I/II<br>III/IV	0.551<br>(0.481–0.622)	0.544<br>(0.468–0.621)	0.444<br>(0.343–0.544)	0.507<br>(0.395–0.620)
Tumor encapsulation	Yes<br>No	0.533<br>(0.462–0.604)	0.529<br>(0.453–0.604)	0.441<br>(0.341–0.541)	0.546<br>(0.435–0.658)
Tumor size (cm)	≤ 5.0<br>> 5.0	0.582<br>(0.512–0.652)	0.654<br>(0.581–0.728)	0.537<br>(0.437–0.637)	0.685<br>(0.578–0.793)
TNM stage	I<br>II/III	0.533<br>(0.462–0.604)	0.616<br>(0.541–0.691)	0.591<br>(0.493–0.688)	0.641<br>(0.531–0.752)
BCLC stage	0/A<br>B/C	0.611<br>(0.541–0.680)	0.651<br>(0.581–0.722)	0.564<br>(0.464–0.663)	0.709<br>(0.610–0.808)
MLR	≤ 0.3<br>> 0.3	0.569<br>(0.499–0.640)	0.620<br>(0.547–0.694)	0.657<br>(0.564–0.751)	0.626<br>(0.515–0.736)
NLR	≤ 2.5<br>> 2.5	0.551<br>(0.480–0.622)	0.613<br>(0.540–0.685)	0.700<br>(0.610–0.789)	0.622<br>(0.510–0.733)
NMLR	≤ 1.2<br>> 1.2	0.593<br>(0.523–0.663)	0.700<br>(0.631–0.769)	0.733<br>(0.648–0.819)	0.691<br>(0.583–0.798)
iMLR	≤ 1.1<br>> 1.1	0.620<br>(0.551–0.690)	0.574<br>(0.500–0.648)	0.686<br>(0.593–0.779)	0.642<br>(0.538–0.747)
Combination of NMLR and iMLR	both low<br>both high	0.646<br>(0.579–0.714)	0.723<br>(0.656–0.789)	0.818<br>(0.746–0.889)	0.755<br>(0.665–0.845)

Abbreviations: RFS: recurrence-free survival; OS: overall survival; NMLR: neutrophil and monocyte to lymphocyte ratio; MLR: monocyte to lymphocyte ratio; NLR: neutrophil to lymphocyte ratio; iMLR: intratumoral CD16/CD8 ratio; AFP: alpha fetoprotein; TNM: tumor node metastasis; BCLC: Barcelona clinic liver cancer.
